# Genome diversification in globally distributed novel marine Proteobacteria is linked to environmental adaptation

**DOI:** 10.1038/s41396-020-0669-4

**Published:** 2020-05-11

**Authors:** Zhichao Zhou, Patricia Q. Tran, Kristopher Kieft, Karthik Anantharaman

**Affiliations:** 1 Department of Bacteriology, University of Wisconsin–Madison, Madison, WI 53706 USA; 2 Department of Integrative Biology, University of Wisconsin–Madison, Madison, WI 53706 USA

**Keywords:** Microbial ecology, Ecosystem ecology

## Abstract

Proteobacteria constitute one of the most diverse and abundant groups of microbes on Earth. In productive marine environments like deep-sea hydrothermal systems, Proteobacteria are implicated in autotrophy coupled to sulfur, methane, and hydrogen oxidation, sulfate reduction, and denitrification. Beyond chemoautotrophy, little is known about the ecological significance of poorly studied Proteobacteria lineages that are globally distributed and active in hydrothermal systems. Here we apply multi-omics to characterize 51 metagenome-assembled genomes from three hydrothermal vent plumes in the Pacific and Atlantic Oceans that are affiliated with nine Proteobacteria lineages. Metabolic analyses revealed these organisms to contain a diverse functional repertoire including chemolithotrophic ability to utilize sulfur and C_1_ compounds, and chemoorganotrophic ability to utilize environment-derived fatty acids, aromatics, carbohydrates, and peptides. Comparative genomics with marine and terrestrial microbiomes suggests that lineage-associated functional traits could explain niche specificity. Our results shed light on the ecological functions and metabolic strategies of novel Proteobacteria in hydrothermal systems and beyond, and highlight the relationship between genome diversification and environmental adaptation.

## Introduction

Proteobacteria constitute one of the most diverse microbial phyla and are successful in most biomes on Earth [1, 2]. Proteobacteria are abundant from pole to pole in the world’s oceans [3, 4], and also from the surface to the deep oceans in vertical cross-sections [5, 6]. Proteobacteria display an enormous functional repertoire and comprise phototrophs, autotrophs, and heterotrophs. In surface oceans, heterotrophic Proteobacteria such as SAR11, SAR86, and Roseobacter are abundant and successful bacterioplankton lineages, which mainly rely on the availability of dissolved organic matter [7]. In dark oceans, Proteobacteria drive carbon cycling through primary production associated with sulfur and methane oxidation [8], as well as heterotrophy [9]. Given their abundance across marine environments and their wide range of metabolic traits, Proteobacteria represent an ideal lineage to investigate links between genome diversification and environmental adaptation. To address this question in a specific environment, we first studied the distribution, metabolism, activity, and ecology of Proteobacteria in deep-sea hydrothermal plumes, a system characterized by the presence of natural geochemical gradients.

Hydrothermal plumes are formed when hot fluids (up to 400 °C) emanate from deep-sea hydrothermal vents and mix with cold deep ocean waters (2–4 °C). This process causes steep thermal and chemical gradients at small spatial scales, and biotic and abiotic processes leading to the formation of a variety of ecological niches that can be exploited by microorganisms [10–12]. Hydrothermal fluids typically entrain substantial concentrations of reduced chemicals and substrates, e.g., hydrogen (H_2_), methane (CH_4_), hydrogen sulfide (H_2_S), ammonia (NH_3_), methanol, C_1_ compounds (formaldehyde, formate, and carbon monoxide), hydrocarbons, and metals (Fe, Mn, As, etc) [13–21]. In chemosynthetic environments of the oceans such as hydrothermal systems, proteobacterial groups actively participate in primary production by utilizing a wide variety of reduced substrates [11, 22]. Specific examples include diverse and active populations of *Sulfurimonas* and *Sulfurovum* (Epsilonbacteraeota) species that oxidize reduced sulfur compounds; *Thioglobus/*SUP05 and *Beggiatoa* (Gammaproteobacteria) that oxidize reduced sulfur compounds and hydrogen for energy generation [11]; *Methylophaga* and Methylococcaceae (Gammaproteobacteria) that can oxidize methane, methanol, and hydrocarbons [23]; and *Hydrogenovibrio* (Gammaproteobacteria) that can oxidize hydrogen and reduced sulfur compounds [24–26]. Finally, given the presence of large fractions of hypothetical proteins in microbial genomes [27–29], it is likely that new enzymatic pathways and microorganisms metabolizing reduced compounds, such as hydrogen and sulfur remain to be discovered [27–29].

In host–microbe systems, typically, proteobacterial endosymbionts (mostly Gammaproteobacteria) of tubeworms can oxidize reduced sulfur species [30], while proteobacterial endosymbionts of bivalves can perform oxidation of reduced sulfur, methane, hydrogen, and carbon monoxide [30–32]. Beyond these host animals, little is known about whether other microbes could also utilize organic compounds from vent-derived chemosynthesis [10]. Organisms in deep-sea systems are often versatile and can exhibit mixotrophic characteristics. Organic carbon from primary production may be used in heterotrophy in hydrothermal plumes as they disperse or be consumed locally. Given the abundance of carbon fixation processes in hydrothermal systems, most research has focused on microbial chemoautotrophy, therefore microorganisms associated with heterotrophy in plumes remain little-studied.

In this study, we reconstructed 51 novel Proteobacteria genomes from the deep-sea hydrothermal plumes and surrounding background seawaters at three distinct locations. These novel Proteobacteria genomes represent nine poorly-studied lineages within Proteobacteria. Metatranscriptomics-derived measurements enabled us to study the activity of these Proteobacteria across a range of environments within and between different plumes and deep ocean samples. The omics-based functional characterization provides insights into organic carbon metabolism, energy transformations, and adaptive strategies in hydrothermal vent ecosystems and beyond. These Proteobacteria lineages have a widespread distribution and can be observed outside of marine environments including freshwaters and the terrestrial subsurface. Overall, our study reveals that genome diversification in globally prevalent and abundant Proteobacteria is associated with environmental adaptation and suggests that the distribution of functional traits could explain their niche-adapting mechanisms.

## Materials and methods

### Sampling, metagenome sequencing, and data processing

The hydrothermal vent plume and background samples were acquired from the following cruises: R/V *New Horizon* to Guaymas Basin (July 2004), R/V *Atlantis* to Mid-Cayman Rise (Jan 2012 and Jun 2013) for Cayman Deep (*Piccard*) and Shallow (*Von Damm*), and R/V *Thomas G Thompson* to the Eastern Lau Spreading Center (ELSC) (May–Jul 2009). Sampling details, and geographic and oceanographic environmental settings are provided elsewhere [10, 33, 34]. In brief, plume and seawater samples were collected either by the Suspended Particulate Rosette (SUPR) filtration device mounted to the remotely operated vehicle or CTD-Rosette bottles [33], and the filters (0.2 μm pore size) were preserved for microbial biomass collection. Two sample processing techniques were employed on our samples from Guaymas Basin and Mid-Cayman Rise, respectively due to advancements in sampling and in situ fixation procedures. First, samples from the Mid-Cayman Rise were collected using the SUPR v2 filtration system and sampler [33] that allowed for in situ fixation using RNA later. On deck, these samples were transferred and stored immediately at −80 °C. Second, samples from the Guaymas Basin were filtered shipboard, preserved immediately in RNA later and stored at −80 °C. Samples collected with the CTD-rosette typically take 30 min to 1 h to be brought up to the surface onboard. These samples were kept in cold and dark conditions, similar to in situ conditions during the process of bringing them up to the deck. DNA (for metagenomics) and cDNA (reverse transcribed from RNA) were sequenced by the Illumina HiSeq 2000 platform (for additional details refer to literature [10, 33–35]). Quality control of raw reads (QC) was performed by Trim Galore within metaWRAP v0.8.6 [36] using default settings.

### Metagenomic binning and genome refinement

MetaSPAdes v3.12.0 [37] was used to assemble QC-passed reads with the settings as follows “–meta -k 21,33,55,77,99”. The QC-passed reads from the individual hydrothermal sites were combined and assembled. For samples from Mid-Cayman Rise, MEGAHIT v1.1.2 [38] was used for the assembly (MetaSPAdes could not be run due to memory size limitations) with the following parameters “–k-list 21,33,55,77,99 -m 0.95”. The resulting assemblies (min scaffold length ≥ 1 kb) and QC-passed reads were used for metagenomic binning by the deep learning algorithm MetaGen [39], and metaWRAP v0.8.6 [36] with self-implemented MaxBin2 [40], metaBAT [41], and metaBAT2 [42] binning modules. Finally, all four sets of Metagenome-assembled genomes (MAGs) were pooled together and subjected to bin dereplication, aggregation and scoring by DAS-Tool with the setting “–score_threshold 0.4” [43].

Resulting MAGs with genome completeness >50% and contamination <10% were further subjected to bin refinement to screen heterogeneous scaffolds potentially originated from contamination and erroneous 16S rRNA sequences using RefineM v0.0.24 [44]. Additional refinement was conducted using VizBin [45] by manually picking scaffolds that are clustered by sequence coverage and 5-nucleotide kmer patterns.

### Distribution of Proteobacteria

To identify the distribution of proteobacterial groups across different marine and terrestrial environments, we used a homology-based identification approach using 16S rRNA gene sequences. From each proteobacterial group of interest, the longest 16S rRNA gene sequences were selected and used for comparison using BLAST (E-value < 1e−5) [46] against metagenomes in the Integrated Microbial Genomes and Metagenomes database (IMG/M) DOE metagenome database [47]. The BLAST hits with the indicated sequence identity within the following taxonomic thresholds were retained (family: 86.5%, order: 82.0%, class: 78.5%, and phylum: 75.0% for full 16S rRNA gene sequence) [48]. The IMG metagenome geographic and environmental details were parsed out and used to make the plots accordingly (R packages: “ggplot2”, “ggmap”, “maps”, and “mapdata”).

### Phylogenetic reconstruction and genome characteristics

The 16 ribosomal proteins (RP) L14, L15, L16, L18, L22, L24, L2, L3, L4, L5, L6, S10, S17, S19, S3 and S8 [49] were identified using HMMER v3.2.1 using NC noise cutoffs [50] and protein sequences were individually aligned with MAFFT v7.271 with default settings [51]. Subsequently, concatenated ribosomal protein alignments of all 16 ribosomal proteins were used for phylogenetic tree reconstruction (RP16 tree, hereafter) by IQ-TREE v1.6.9 [52] with “-m MFP -bb 100 -s -redo -mset WAG,LG,JTT,Dayhoff -mrate E,I,G,I + G -mfreq FU -wbtl” settings. The RP16 tree topology resolved from this study was compared to that from Genome Taxonomy Database (GTDB) [53] to confirm the phylogenetic position of these novel Proteobacteria groups. Genome characteristics were provided by various programs, including (1) genome phylogeny (GTDB, NCBI and manually-curated ones) using GTDB-Tk v0.1.3, (2) genome coverage, completeness and contamination, and strain heterogeneity (by CheckM [54]), (3) 16S rRNA phylogeny and genome characteristics (Ones with incongruent taxonomy to that of RP16 tree were filtered), and (4) tRNA statistics (by tRNAscan-SE v2.0 [55]). The 16S rRNA gene phylogenetic tree was reconstructed using IQ-TREE v1.6.9 with “-st DNA -m GTR + G4 + F -bb 1000 -alrt 1000” settings.

### Metabolic gene annotations

We applied the biogeochemical functional trait profiler METABOLIC v1.3 on reconstructed genomes [56]. Hmmsearch was used to scan for potential metabolic genes from MAGs using manually curated noise cutoffs. We applied manually curated trusted cutoffs (-TC option) for scanning HMMs against sulfur cycling genes. KEGG Orthology (KO) [57] annotation was conducted using GhostKOALA (accessed 04-22-2019) [58], KAAS (accessed 04-22-2019-04-22) [59], and EggNOG emapper v4.5.1 [60]. The KO ID was assigned to a protein in the following the order (1) GhostKOALA KO, (2) KAAS KO, (3) EggNOG emapper KO, and (4) EggNOG emapper COG transferred KO. In addition, we also utilized the NCBI-nr database (Jun 2018 release) to annotate proteins using DIAMOND BLASTP (DIAMOND v0.9.24) [61]. If all five annotation approaches produced no annotations, we assigned “N/A” to this protein.

### Genome functional profiles and comparative genomic analysis

All genomes were dereplicated using dRep v2.3.2 [62] and only genomes with over 80% genome completeness, <10% genome redundancy were used (except for some lineages with limited genomes, e.g., for SAR86 and Hyrcanianaceae; we also included several genomes with ~70% completeness). METABOLIC v1.3 was used to assign functions. The assignment of a KEGG module to a proteobacterial group was conducted by first assigning the existence of individual KEGG module components (the cutoff value for existence in group members was 50%), and subsequently assigning the presence of entire KEGG modules by presence of all the compositional KOs (cutoff value was 75%). We clustered proteins of all genomes into ortholog groups (OGs) using OrthoFinder v2.2.7 [63]. The comparative genomic analysis was conducted by sorting the distribution of OG among different clades.

### Annotation of carbohydrate-active enzymes and peptidases

Proteins were identified by hmmscan using the dbCAN2 database [64] (dbCAN-HMMdb-V7) for annotating carbohydrate-active enzymes (CAZymes). Only the glycoside hydrolase and polysaccharide lyase annotations were retained. Peptidase (also including peptidase inhibitor) annotation was conducted by using DIAMOND BLASTP to search against the MEROPS database (pepunit dataset, accessed 04-22-2019) [65] with “-k 1 -e 1e-10 –subject-cover 80 –id 50” settings.

### Metagenomic and metatranscriptomic analysis

The quality control-passed metagenomic reads were used for mapping against MAGs using Bowtie 2 v2.3.4.1 [66] with default settings. The normalized genome coverage was calculated by using the average coverage for all scaffolds and normalizing it with 100M reads per metagenomic dataset. QC-passed and rRNA-filtered (conducted by SortMeRNA with default settings [67]) metatranscriptomic reads were mapped against each gene. The metric Reads Per Kilobase per Million mapped reads (RPKM) was calculated accordingly. To compare the expression level of individual genes in genomes from difference environments, we also normalized RPKM values by dividing them with corresponding genome coverage to normalize for the influence of different genome sequencing depth in each environment.

## Results

### Reconstruction of genomes from deep-sea hydrothermal plumes

Hydrothermal vent plume and background deep-sea samples were acquired during the following cruises: R/V New Horizon to Guaymas Basin (July 2004), R/V Atlantis to Mid-Cayman Rise (Jan 2012 and Jun 2013) for Cayman Deep (*Piccard*) and Shallow (*Von Damm*) plume and background seawater samples, and R/V Thomas G Thompson to the Lau Basin (May–Jul 2009) for both plume and background seawater samples. Details of sample collection, preservation, and DNA/RNA extraction and processing are described in detail elsewhere [10, 33, 34].

**Fig. 1:**
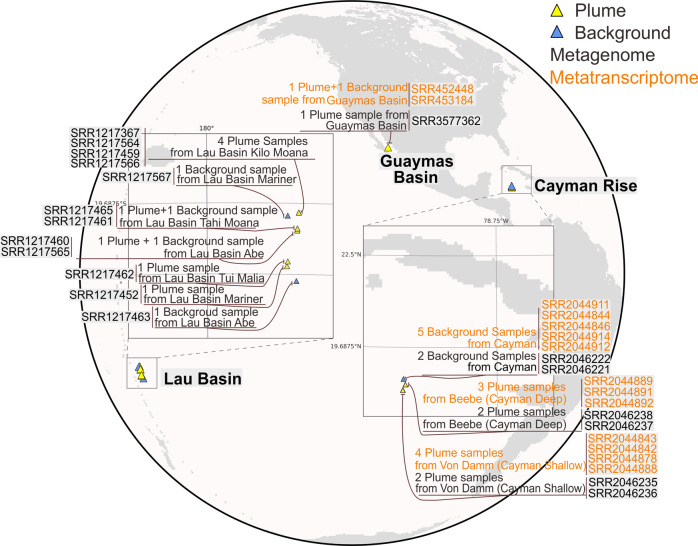
Schematic map representing the sampling locations of hydrothermal samples. The retrieved metagenomic datasets include one from Guaymas Basin, twelve from Lau Basin, and six from Mid-Cayman Rise. Detailed sample, metagenome, and metatranscriptome information is labeled.

In this study, we reconstructed genomes from publicly available shotgun metagenomic sequencing datasets from 19 deep-sea hydrothermal plume and surrounding background seawater samples from Guaymas Basin (Guaymas), Mid-Cayman Rise (Cayman) and Lau Basin (Lau) (Fig. 1). Additionally, we analyzed the 14 metatranscriptomic datasets that were paired with metagenomics samples from Guaymas and Cayman (Supplementary Table S1). Following quality-control, filtered reads were used to assemble scaffolds *de novo* according to the location of metagenomic samples. Metagenomic binning resulted in 250 metagenome-assembled genomes (MAGs) which have genome completeness >50% and genome contamination <10% in accordance with previously suggested Minimum Information about a Metagenome-Assembled Genome (MIMAG) standards [68].

### Phylogeny and identification of proteobacterial lineages

To identify the taxonomy of the reconstructed genomes, we used a comprehensive phylogenetic approach. First, we constructed a phylogenetic tree using a set of concatenated 16 ribosomal proteins (RP16 tree). The reconstructed phylogenetic tree revealed that bacteria comprised 219 of the 250 genomes, with 92 of them from the group Proteobacteria. We then conducted detailed taxonomic curation of all reconstructed genomes by comparison with specific databases and phylogenetic trees, namely NCBI, GTDB, and RP16. A companion 16S rRNA gene phylogenetic tree (using genes retrieved from MAGs) also has the congruent phylogeny (Supplementary Fig. S1). Of the 92 Proteobacteria genomes, we determined 51 to be phylogenetically novel (Supplementary Table S2); all lack a defined taxonomy at the scale of family, order, and/or phylum, coupled with a lack of understanding of their metabolism and ecology.

**Fig. 2:**
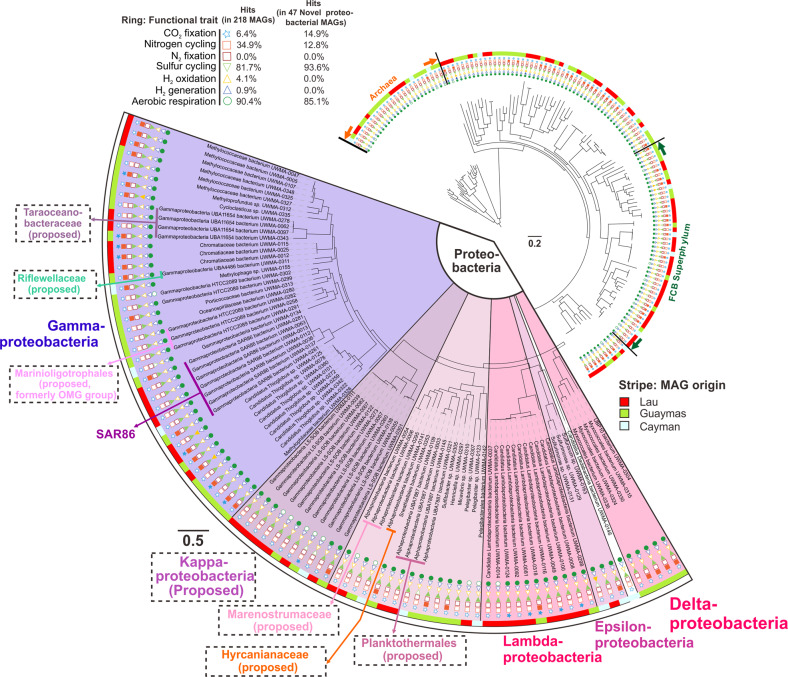
Phylogenetic tree of the hydrothermal plume and deep-sea-derived MAGs based on the concatenated 16 ribosomal protein alignment (RP16 tree). Functional traits associated with carbon, nitrogen, sulfur, hydrogen cycling, and oxygen respiration are shown. Filled and unfilled circles denote the presence/absence of function traits within a genome. This tree was visualized using iTOL [96] (https://itol.embl.de/). This figure does not necessarily reflect the branching order of all representative Proteobacteria groups.

Based on the RP16 tree, we classified and defined nine proteobacterial lineages at different levels, including two phyla, three classes and four families (Fig. 2 and Supplementary Figs. S2, S3). We propose the names Marenostrumaceae for UBA2165 (since the UBA2165 type strain was first reconstructed from the Mediterranean Sea [44]; known as “Mare Nostrum” in Latin); Hyrcanianaceae for group Casp-alpha2 (since the Casp-alpha2 type strain was first reconstructed from the Caspian Sea [44]; known as the “Hyrcanian Ocean” in ancient Greek), Taraoceanobacteraceae for UBA11654, which was first reconstructed from Tara Ocean metagenome datasets [69]; Riflewellaceae for UBA4486 which was first described from terrestrial aquifer wells at Rifle, Colorado, USA [49]; Marinioligotrophales for the formerly OMG bacteria, the Oligotrophic Marine Gammaproteobacteria [70]; Planktothermales for UBA7887, which are reconstructed from hydrothermal plume environments [44]; and Kappaproteobacteria for the former LS-SOB group [44], which are ubiquitous in coastal systems and the ocean water column. This classification was also supported by the taxonomic tree associated with the GTDB taxonomy database [53]. Finally, Lambdaproteobacteria were recently described and characterized from a groundwater ecosystem [49], while this is the first study to retrieve and study genomes recovered from deep-sea hydrothermal plumes or any marine environment for this newly-discovered phylum. Most of the Proteobacteria genomes from this study have metabolic capacities associated with aerobic respiration, sulfur cycling, and CO_2_ fixation (Fig. 2 and Supplementary Table S3).

### Distribution and abundance

Novel Proteobacteria genomes identified by us constituted 23% of all reconstructed genomes from the hydrothermal environments studied here. Yet, analyses of genome coverage indicated that they comprised 36% of the microbial community, which suggests they are relatively more abundant than others (Supplementary Table S4). To determine if this abundance of Proteobacteria also translated to a higher activity in hydrothermal plumes, we compared gene expression of these organisms with members of other dominant marine phyla such as Chloroflexi (SAR202) and Bacteroidetes (Supplementary Table S4). All comparisons of metatranscriptomic mapping were conducted at the resolution of microbial groups (not individual MAGs) between novel Proteobacteria and other microbial groups. The proportion of gene expression by novel Proteobacteria was higher than that of other phyla, suggesting that novel Proteobacteria were relatively more active within community.

**Fig. 3:**
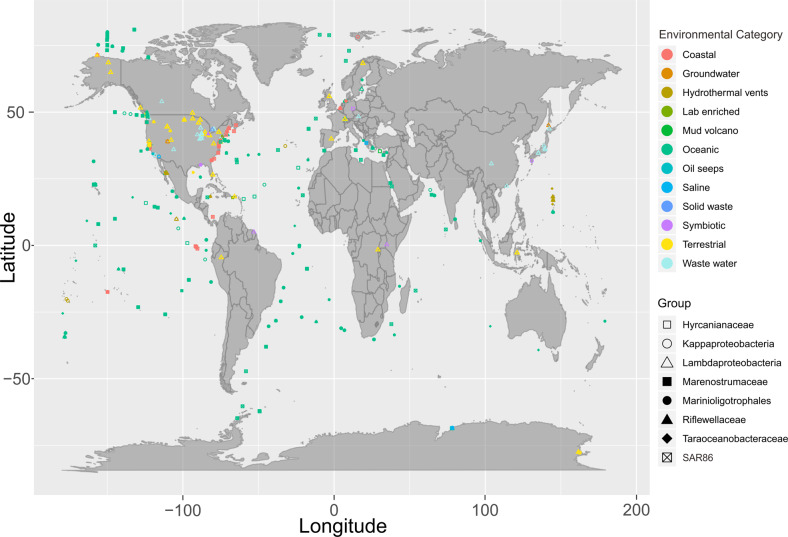
World map showing the distribution of novel Proteobacteria lineages. Environmental categories were parsed and summarized from “ecosystem type” information from all investigated metagenomes.

We then assessed the global distribution of these nine Proteobacteria lineages by examining their presence in metagenomes from the IMG/M DOE database [47]. While most of these Proteobacteria lineages were widely distributed, specifically Kappaproteobacteria, SAR86, and Marinioligotrophales were especially abundant and distributed worldwide in oceanic and coastal environments (Fig. 3). In addition, these groups were also observed in other environments outside of marine systems such as in association with a host (symbiosis), terrestrial environments, and engineered systems. Members of these Proteobacteria lineages that are abundant and active in deep-sea hydrothermal systems, are well adapted to the ambient environments and can have important ecological impacts. Finally, we used this 16S rRNA-based community survey to identify Proteobacteria lineages undergoing genome diversification and conducted comparative genomics to delineate their adaptation to specific environments.

### Central metabolism and respiration

**Fig. 4:**
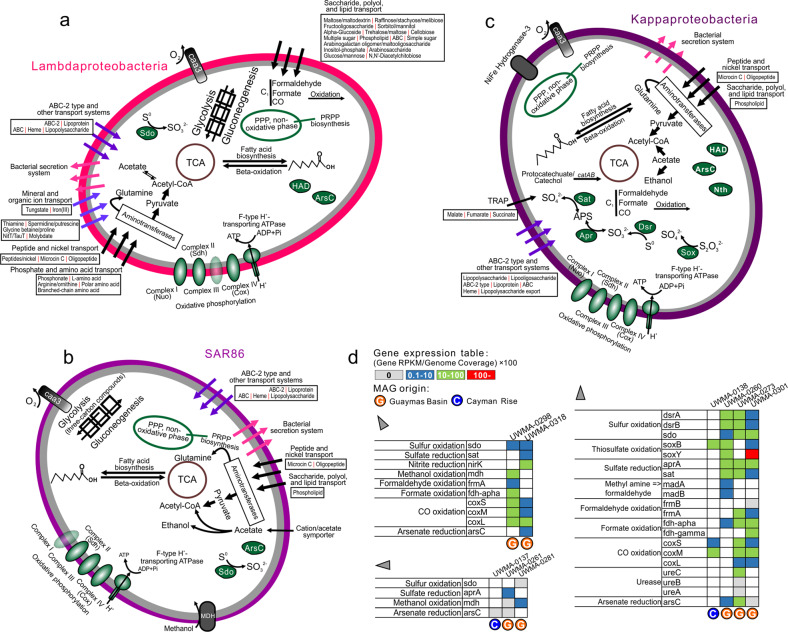
Cellular maps of inferred metabolic capacities and activities of Lambdaproteobacteria, SAR86, and Kappaproteobacteria. **a**–**c** Organismal profiles of metabolic capacity for Lambdaproteobacteria, Kappaproteobacteria, and SAR86, respectively. Items in gray indicate traits that are not present in over 50% of sampled genomes. **d** Tables indicating the normalized gene expression level of important pathways associated with energy metabolism in novel Proteobacteria genomes.

**Fig. 5:**
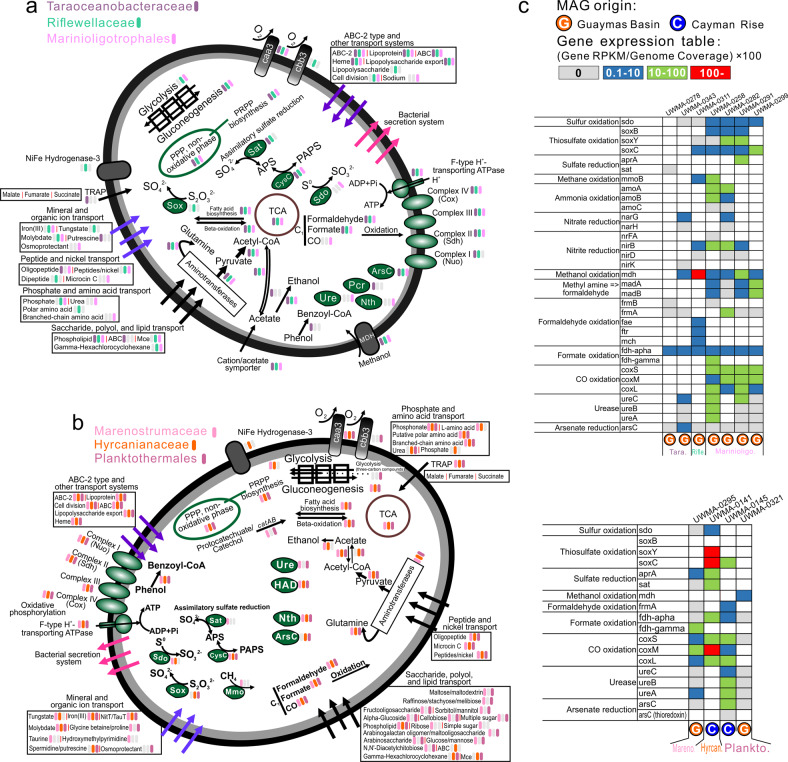
Cellular maps of inferred metabolic capacities and activities of the six Proteobacteria families and orders. **a** Organismal profiles of metabolic capacities for Taraoceanobacteraceae, Riflewellaceae, Marinioligotrophales, **b** Organismal profiles of metabolic capacities for Marenostrumaceae, Hyrcanianaceae, Planktothermales. Items in gray indicate traits that are not present in over 50% of sampled genomes. **c** Tables indicating the normalized gene expression level of important pathways associated with energy metabolism in novel Proteobacteria genomes.

We used reconstructed MAGs from this study and publicly available genomes with completeness over 80% to reflect the general metabolic and functional capacities of novel Proteobacteria (Supplementary Tables S5, S6). In addition, the metatranscriptomic datasets from Guaymas and Cayman enabled investigation of metabolic gene expression patterns (Figs. 4, 5 and Supplementary Table S6).

All nine proteobacterial lineages contained genes for central carbon metabolism pathways for biosynthesis and energy transfer: citric acid cycle (TCA cycle), glycolysis (some could only metabolize three-carbon compounds) and gluconeogenesis, peptide and amino acid utilization, pentose phosphate (PPP) pathway and PRPP biosynthesis for the generation of some nucleotide and amino acid precursors, and fatty acid biosynthesis and beta-oxidation (Figs. 4, 5 and Supplementary Table S7). These proteobacterial lineages contain either caa3/cbb3 type cytochrome *c* oxidases for aerobic respiration and nearly a complete set of complexes of oxidative phosphorylation for ATP generation, suggesting that fermentation and respiration can both take place.

### Organic carbon metabolism

We investigated organic carbon metabolism in novel Proteobacteria by identifying the presence of transporters, secretion systems, CAZymes [71], and peptidases in the genomes. Functional predictions of cell membrane transport and secretion systems indicate that Lambdaproteobacteria, Marenostrumaceae, and Planktothermales have more transporters involved with monosaccharides, polysaccharides, polyols, and lipids which may be used as organic nutrients. Moreover, Kappaproteobacteria, Taraoceanobacteraceae, Marenostrumaceae, Hyrcanianaceae, and Planktothermales contain tripartite ATP-independent periplasmic transporters for transporting C_4_-dicarboxylates [72] while SAR86, Taraoceanobacteraceae, Riflewellaceae, and Marinioligotrophales contain cation/acetate symporters for direct acetate incorporation (Figs. 4, 5). Our analyses indicate that many of these organisms are capable of fermentation from acetate to ethanol and have wide-ranging capacities for the oxidation of C_1_ compounds such as formaldehyde, formate, and carbon monoxide. Meanwhile, Kappaproteobacteria, Taraoceanobacteraceae, Marenostrumaceae, Hyrcanianaceae, and Planktothermales can also degrade and utilize aromatic compounds, such as phenol and protocatechuate/catechol (Figs. 4, 5). Lambdaproteobacteria, Kappaproteobacteria, Marinioligotrophales, Marenostrumaceae, Hyrcanianaceae and Planktothermales contain organisms that can actively oxidize C_1_ compounds (only formaldehyde, formate, and carbon monoxide) from hydrothermal environments. Lambdaproteobacteria, SAR86, Taraoceanobacteraceae, Riflewellaceae, Marinioligotrophales and Planktothermales demonstrated high expression levels of methanol oxidation encoding genes. The highest expression level was observed in Riflewellaceae organisms (Figs. 4, 5). Genomes from Kappaproteobacteria and Marinioligotrophales encoded highly active genes for the utilization of methyl amines. These results indicate that these Proteobacteria are well adapted to hydrothermal ecosystems, and their metabolic activities are connected to the transformation of organic compounds of hydrothermal origin which are entrained in the plume, such as C_1_ (formate, formaldehyde, carbon monoxide) [13–16] and methylated compounds (methanol and methylamine) [17, 18].

**Fig. 6:**
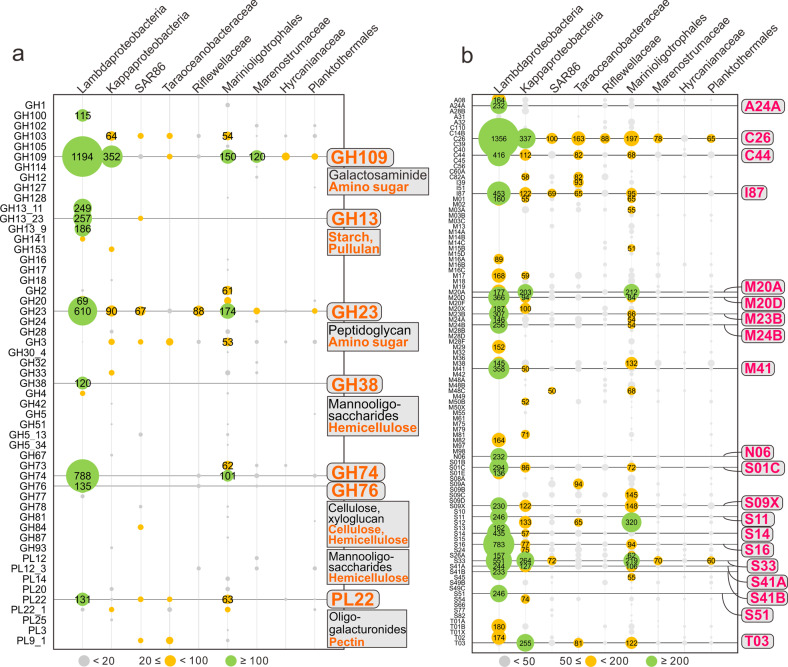
Coverage profiles of carbohydrate-active enzymes (CAZymes) and peptidases in novel Proteobacteria genomes. **a** Glycoside Hydrolase (GH) and Polysaccharide Lyase (PL) gene coverage was calculated by multiplying the identified number of CAZymes with normalized genome coverage. **b** Peptidase (also includes peptidase inhibitors) gene coverage was calculated by multiplying the identified number of peptidases with normalized genome coverage.

**Fig. 7:**
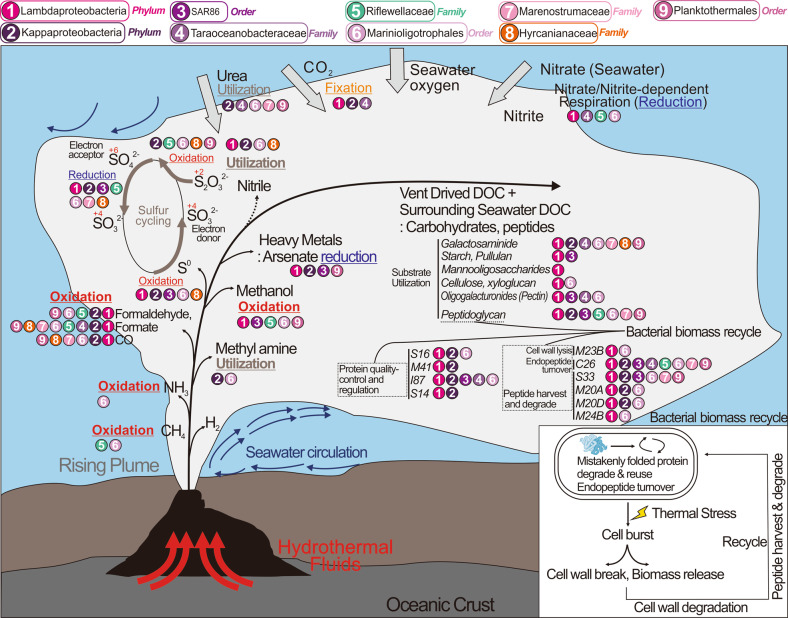
Conceptual representation of novel Proteobacteria and their ecological roles during the development and dispersal of hydrothermal plumes and the deep-sea. Ecological roles described here drive genome diversification. Hydrothermal plumes are shown to be distinct from the deep ocean water column. This distinction arises from temperature and density gradients of fluid masses. Only traits associated with biogeochemical cycling and energy metabolism are shown.

To study the potential of novel Proteobacteria to breakdown carbohydrates and proteins, we screened all genomes for the presence of CAZymes and peptidases. Patterns of normalized CAZyme and peptidase gene coverage demonstrate that Lambdaproteobacteria, Kappaproteobacteria, and Marinioligotrophales are involved in carbohydrate and protein scavenging (Figs. 6, 7). Gene expression profiles suggest that Lambdaproteobacteria, Kappaproteobacteria, and Marinioligotrophales CAZymes and peptidases are highly active in Guaymas and Cayman (Supplementary Tables S9, S10). GH109 (α-N-acetylgalactosaminidase) for degradation of amino sugars, GH23 (lysozyme) for degradation of peptidoglycans and PL22 (oligogalacturonide lyase) for degradation of oligogalacturonides (a product of pectin degradation) are widely distributed in the Proteobacteria. In contrast, other families have limited distributions in specific lineages. For example, GH13 (α-amylase) for degradation of starch and pullulans, GH38 (α-mannosidase) and GH76 (ß-mannosidase) for degradation of mannooligosaccharides, and GH74 (ß-1,4-endoglucanase, endoglucanase) for degradation of cellulose and xyloglucan are primarily distributed in Lambdaproteobacteria and highly expressed. The other five proteobacterial lineages (SAR86, Taraoceanobacteraceae, Riflewellaceae, Hyrcanianaceae, and Planktothermales) exhibited a limited distribution of CAZymes and peptidases suggesting little to no involvement in carbohydrate and protein breakdown. Overall, this indicates that novel Proteobacteria participate in carbon and energy cycling in hydrothermal environments with differing metabolic strategies, which are mainly reflected in their divergent organotrophic capacities.

Lambdaproteobacteria genomes encoded the highest abundance of peptidases (Figs. 6, 7). The most abundant and actively expressed peptidases were related to protein quality control and regulation, e.g., S16 (Lon-A peptidase), an unfolded protein degrader, M41 (FtsH peptidase), and I87 (FtsH inhibitor), which inhibit FtsH and modulate the degradation of mistranslation products that disrupt membranes, and S14 (Clp peptidase), which regulates specific protein degradation (Fig. 6 and Supplementary Table S9). This could result from stress response activities that may be induced from association with high-temperature fluids [73] and serve as a protective and regulatory mechanism for cell membrane maintenance and protein transformations. Besides, there are also other abundant and active endo/extracellular peptidases in Lambdaproteobacteria which are responsible for harvesting and degrading peptides and endopeptide turnover, e.g., C26 (gamma-glutamyl hydrolase) for the turnover of folyl poly-gamma-glutamates, S33 (prolyl aminopeptidase), an extracellular peptidase prone to proline-rich substrate utilization, M20A and M20D (glutamate carboxypeptidase), and M23B (ß-lytic metallopeptidase) which lyse cell walls.

### Nitrogen and sulfur metabolism

Kappaproteobacteria, Taraoceanobacteraceae, Marinioligotrophales, Marenostrumaceae, and Planktothermales genomes encode for ureases that are highly expressed suggesting that urea may be a common source of nitrogen. Nitrogen cycling activities by Proteobacteria involve both the oxidative and reductive cycle of nitrogen. Taraoceanobacteraceae, Riflewellaceae, and Marinioligotrophales encode genes associated with ammonia oxidation (*amoABC*) and nitrite/nitrate reduction (Figs. 4, 5). No single organism possessed the entire complement of genes for denitrification, significantly no genes were observed for nitric and nitrous oxide reduction in any genomes. Only genes for the membrane-bound Nar proteins for nitrate reduction were observed, no periplasmic Nap genes for nitrate reduction were observed in any genomes. Genes for nitrite reduction included the copper-containing *nirK* (for reduction to nitric oxide) and *nirBD* and *nrfA* for dissimilatory reduction of nitrite to ammonia. Many of the Proteobacteria were associated with sulfur cycling comprising the oxidative cycle of sulfur. All proteobacterial genomes lacked genes for the oxidation of hydrogen sulfide to sulfur. However, many of them encode genes for sulfur dioxygenases and reverse-dissimilatory sulfite reductases for sulfur oxidation. In particular, Kappaproteobacteria genomes encode highly expressed genes for complete oxidation of sulfur to sulfate including the *dsrAB* for the oxidation of elemental sulfur to sulfite and *aprAB* and *sat* for the oxidation of sulfite to sulfate (Supplementary Figs. S4, S5, and Supplementary Table S10). Kappaproteobacteria, Riflewellaceae, Hyrcanianaceae, and Planktothermales genomes possessed the Sox enzyme complex for the utilization of thiosulfate. We investigated the presence of the *soxCD* genes in all genomes for complete oxidation of thiosulfate in lieu of disproportionation. Amongst these, Kappaproteobacteria genomes lacked *soxCD* suggesting that they can only disproportionate thiosulfate to elemental sulfur and sulfate while Riflewellaceae, Hyrcanianaceae, and Planktothermales can undertake complete oxidation of thiosulfate to sulfate. Kappaproteobacteria and Hyrcanianaceae genomes exhibited high levels of *sox* gene expression suggesting active utilization of thiosulfate in hydrothermal plumes (Figs. 4, 5 and Supplementary Table S10). Overall, these results suggest that these novel Proteobacteria actively oxidize and cycle various nitrogen and sulfur species as nutrient and energy sources [11].

### Metabolism of iron and arsenic

Genes encoding for mineral transport enzymes for Fe (III) could be found in the Lambdaproteobacteria, Riflewellaceae, Marinioligotrophales, Marenostrumaceae, Hyrcanianaceae and Planktothermales genomes, suggesting that these Proteobacteria likely participate in the acquisition of Fe from precipitating minerals in hydrothermal plumes. Microorganisms store iron within the cell by reducing Fe (III) to Fe (II), and incorporate iron into a variety of organic compounds by forming C–Fe or S–Fe bonds, such as in metalloproteins, ferredoxins, and NADH dehydrogenase which are of significant importance to cellular activities [74]. In doing so, plume Proteobacteria may serve as a part of the microbial Fe pump to scavenge and store mineral-bound Fe in biomass, to sequester Fe in the organic carbon pool after cell death, and to transfer Fe widely as plumes disperse across the oceans [75].

Arsenic and arsenic minerals are discharged in hot, mineralized hydrothermal fluids [76], and organisms in close proximity to the rising plume need to be resistant to elevated arsenic concentrations. Nearly all proteobacterial genomes contained genes for arsenate reduction (*arsC*), and many *arsC* genes have considerably high expression in hydrothermal plumes, such as in Planktothermales at Cayman, and Taraoceanobacteraceae and Kappaproteobacteria at Guaymas. Arsenic resistance and detoxification are mediated by arsC and highly abundant arsC genes have previously been found in hydrothermal systems such as at the iron-rich hydrothermal microbial mats from Lō'ihi Seamount, Hawai’i [77].

### Linking genome diversification to adaptation of functional traits

Microbial traits often evolve in close coordination with their environment. For instance, biogeographical distribution of *Prochlorococcus* suggests that some genome contents are associated with specific regions and environmental factors, such as low nutrients and temperature [78]. Therefore, we investigated genome diversification of individual clades of these nine proteobacterial lineages in the context of niche-specific metabolic functions. We specifically identified proteobacterial lineages from hydrothermal environments that were also observed in other broad marine and terrestrial environments and performed the comparative genomic analyses of these genomes. Amongst the lineages investigated by us, SAR86, Kappaproteobacteria, Muproteobacteria, and Lambdaproteobacteria exhibited the strongest evidence of genome diversification and its association with environmental adaptation.

#### SAR86

**Fig. 8:**
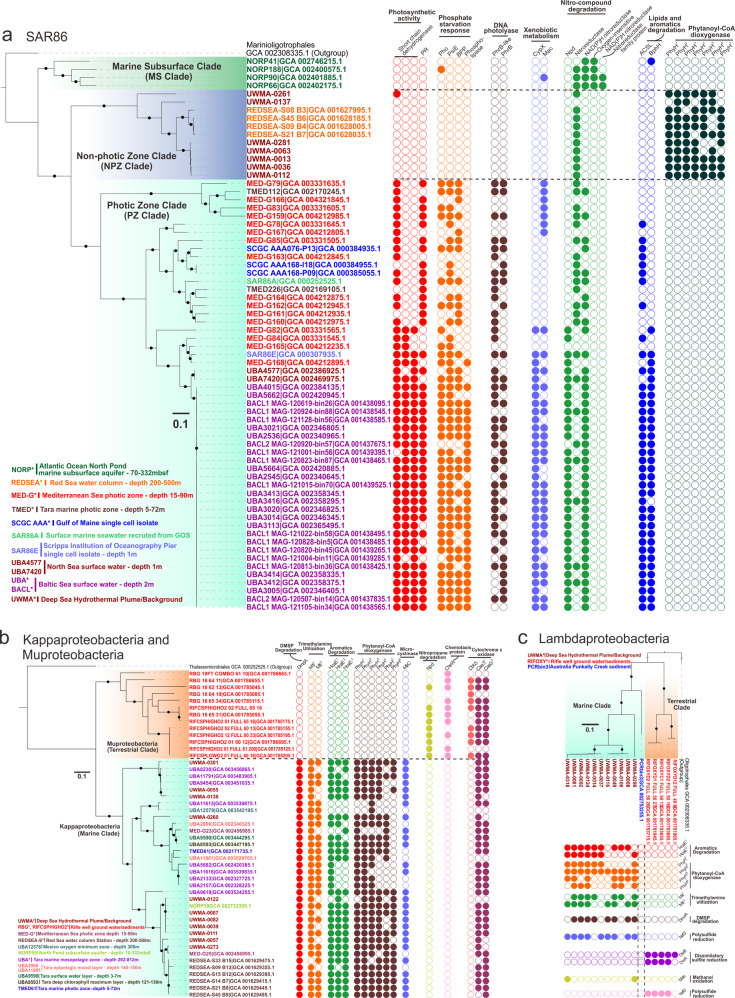
Comparison of lineage-specific protein families associated with environmental adaptation in SAR86, Kappaproteobacteria, Muproteobacteria, and Lambdaproteobacteria populations. Phylogenetic trees were reconstructed based on concatenated 16 RPs, and branches with bootstrap values [ultrafast bootstrap (UFBoot) support values] over 90% by IQ-TREE were labeled with black dots. The tree scale is provided in each sub-panel with 0.1 amino acid substitutions per site, accordingly. Each circle on the panel right to the tree indicates an ortholog group (OG) with a specific function. Filled circles represent the presence and unfilled circles represent the absence of specific traits.

SAR86 is a globally distributed clade of heterotrophic Proteobacteria. Here, we have provided some of the first evidence elucidating the roles and importance of SAR86 in hydrothermal plumes. Our concatenated ribosomal protein-based phylogenetic tree for SAR86 indicated the presence of three distinct clades, namely the non-photic zone clade that also harbors hydrothermal plume derived genomes, the photic zone clade, and the marine subsurface clade (Fig. 8a), which are associated with the specific environments they inhabit. To understand genome diversification associated with this niche differentiation, we examined clade-specific genes and metabolic pathways in these SAR86 clades. All three clades possess a unique distribution of orthologous genes with specific functions that point to their genome diversification (Fig. 8a). The photic zone clade exclusively possesses proteorhodopsins as the light-driven proton pump for energy conservation which is important for their limited chemoorganotrophic lifestyle [79]. In spite of the importance of proteorhodopsin in these organisms, they lack the ability to synthesize retinol as a chromophore [80, 81]; in line with previous reports, we hypothesize that short-chain dehydrogenases are responsible for converting other substrates (e.g., retinal or β-carotene) to retinol [80]. The aquatic β-propeller phytase (BPP) enzymes are found in marine bacteria for the mineralization of phytate to recycle phosphorus [82]. We observed the presence of BPP, phosphate-starvation-inducible protein, alkaline phosphatase [83] and phospholipase only in the photic zone clade suggesting that they are more subjected to a phosphorus-limited environment and have evolved to gain a set of response mechanisms against phosphorus-starvation. Finally, the exclusive distribution of DNA photolyases (PhrB/-like) in the photic zone also indicates that SAR86 in this environment have evolved to repair DNA damage caused by exposure to ultraviolet light [84] (Fig. 8a).

Genes unique to the non-photic zone clade (that includes genomes from hydrothermal environments) of SAR86 include phytanoyl-CoA dioxygenases (PhyH) associated with the breakdown of chlorophyll. The copy number of *phyH* in this clade ranged from 4 to 7. PhyH could hydroxylate the methyl-branched chain of phytanoyl-CoA and is required in the alpha-oxidation pathway of fatty acid metabolism to move the methyl-group from beta position to the alpha position [85]. Together with the metabolism of alcohol and aldehyde dehydrogenases and acyl-CoA synthetase, it is feasible for bacteria to transform phytol (a long-chain alcohol constituent of chlorophyll) to phytanoyl-CoA and pass the products downstream towards beta-oxidation and energy utilization [85, 86]. The exclusive distribution of PhyH in the non-photic zone clade implicates that this specific group could potentially scavenge the degradation products of chlorophyll for carbon and energy demand, which could possibly be of phytoplankton-origin from the upper ocean layers (e.g., deep chlorophyll maximum) [87]. Macrolide transporter and cytochrome P450 are potentially responsible for the degradation of xenobiotics (non-naturally produced compounds toxic to microbes, e.g., therapeutic drugs, antibiotics) [80], and the genes encoding for them are only found in photic zone genomes (Fig. 8a). It also indicates that photic zone genomes possess nitronate monooxygenases and nitroreductases for degrading nitronate and nitro-containing compounds (e.g., nitroaromatics and nitroheterocyclic compounds) (Fig. 8a). Furthermore, photic zone genomes possess additional capacities to degrade long-chain fatty acids and aromatics (Fig. 8a). This potentially indicates that xenobiotic compounds, nitro-compounds, lipids, and aromatics are universal in the surface oceans and surface microbes are adapted for biological defense and substrate utilization. SAR86 inhabiting the marine subsurface are also capable of degrading nitro-compounds, as their genomes contain different types of nitroreductases, such as NAD(P)H dependent, oxygen-insensitive nitroreductases, and other nitroreductase family proteins (Fig. 8a).

#### Kappaproteobacteria and Muproteobacteria

Our genome diversification analysis suggests that two lineages, Kappaproteobacteria and Muproteobacteria evolved to uniquely inhabit specific environments, namely terrestrial (Muproteobacteria) and marine (Kappaproteobacteria). The two lineages are closely related, representing monophyletic deep-branching clades that likely share a common ancestor (Supplementary Fig. S2). Kappaproteobacteria genomes encode important functional traits for utilizing elemental and energy sources in the ocean (Fig. 8b). Dimethyl sulfoniopropionate (DMSP) is a widely distributed marine algal osmolyte and is well known as a significant source of carbon and sulfur for bacterioplankton [88], while trimethylamine (TMA) is part of the oceanic organic nitrogen pool and produced by reduction of many marine osmolytes such as glycine betaine, trimethylamine oxide (TMAO), and choline [89]. Kappaproteobacteria genomes encode enzymes to utilize all these above-mentioned compounds. Similar to SAR86, Kappaproteobacteria can also potentially utilize aromatics and chlorophyll degradation products in the ocean. Furthermore, microcystin, as a group of toxins produced by cyanobacteria, could be found not only in freshwater but also marine environments [90]. Kappaproteobacteria contain genes encoding for microcystinase which could degrade and detoxify microcystin that is generated by marine cyanobacteria [91]. These results suggest that Kappaproteobacteria are well adapted to life in the marine water column.

In contrast, Muproteobacteria genomes were primarily sourced from terrestrial ground water and sediments and were absent in marine environments [49]. Microbes encoding chemotaxis proteins (CheW) are better adapted to sense and respond to the chemical gradients beneficial to their survival in porous underground environments. Nearly all Muproteobacteria genomes we examined possessed CheW. Muproteobacteria also encode genes to utilize nitropropane (a potential industrial hazardous compound) from terrestrial environments (Fig. 8c). Despite their existence in terrestrial environments with limited oxygen, Muproteobacteria possess cytochrome *c* oxidases and other complexes for aerobic respiration (Supplementary Table S11). Muproteobacteria genomes contain both the caa3 type (also referred to as A-type) and cbb3 type (also referred to as C-type) cytochrome *c* oxidases, while Kappaproteobacteria only contain the caa3 type. The cbb3 type cytochrome *c* oxidase has a higher oxygen binding affinity in comparison to caa3 and helps microorganisms to respire under low-oxygen conditions [92]. These results provide a genome-based understanding of Muproteobacteria as a lineage of organisms that are highly adapted to survive and proliferate in the terrestrial subsurface.

#### Lambdaproteobacteria

Similar to Kappaproteobacteria and Muproteobacteria, the marine clade of Lambdaproteobacteria contains genes for degradation of aromatics, chlorophyll degradation products, DMSP, and TMA (Fig. 8). The functional trait of methanol oxidation is essential for utilizing methanol entrained in the hydrothermal plume as indicated (Fig. 8). Less well-known is their role in terrestrial environments and the metabolic differences between the marine and terrestrial clades. Both the terrestrial and marine clades of Lambdaproteobacteria contain genes for the reduction of polysulfide, however, the enzymes belong to different protein families, which suggests that they might operate differently in corresponding environments to deal with polysulfide reduction, e.g., with different substrate binding affinities, different enzymatic activities and/or adaptation to salinity (Fig. 8). Finally, only organisms from the terrestrial clade of Lambdaproteobacteria possess the dissimilatory sulfite reductase pathway to reduce sulfite to hydrogen sulfide [93].

## Discussion

Overall, our research provides the first comprehensive study into the ecological functions and metabolic capacities of nine globally distributed proteobacterial lineages that are abundant and active in deep-sea hydrothermal systems. Specifically, within deep-sea hydrothermal plume environments, our results suggest that organisms from these proteobacterial lineages have versatile metabolisms associated with chemolithotrophic activity and utilization of C_1_ compounds, sulfur and thiosulfate oxidation, and organotrophic activity dependent on fatty acids, aromatics, carbohydrates, and peptidases. All proteobacterial lineages from hydrothermal plume are comprised of organisms that can potentially respire oxygen or nitrate/nitrite based on their genome contents.

Compared to dominant chemolithotrophic activities of the plume microbiome based on sulfur, hydrogen, and methane [28], the metabolic versatility observed in these nine proteobacterial lineages provides new insights on microbial adaptation to changing geochemistry. Bacterial cells in proximity to venting fluids can suffer from high-temperature stress, which can cause protein unfolding [94] and mistranslation [95], and even cell burst. Novel Proteobacteria in this specific environment possess abundant and active peptidases for protein quality control and regulation and enzymes for endopeptide turnover. They also possess enzymes for cell wall lysis, peptide harvest and degradation that use organics in surrounding environments after cell burst. This suggests that they can actively recycle biomass within the cell and in ambient environments under hydrothermal conditions.

Although the novel Proteobacteria genomes discovered in this study were reconstructed from hydrothermal environments, the nine proteobacterial lineages that they represent are distributed worldwide in various environments. Our study highlights the discovery of functional traits that can explain the niche-adapting mechanisms of marine/terrestrial and marine layer divisions. Bacterial genomes from the photic zone exclusively possess proteorhodopsins (for light-based energy generation) and DNA damage repair proteins due to light penetration in surface oceans. Additionally, they are adapted for biological defense and substrate utilization to deal with various compounds in the surface ocean, e.g., xenobiotics, nitro-compounds, lipids, and aromatics. The non-photic zone bacterial genomes including those derived from hydrothermal plumes can potentially scavenge chlorophyll degradation products as carbon and energy sources, which are of phytoplankton-origin from upper marine layers. The division between marine and terrestrial systems can also drive functional divergence. This is highlighted by observations involving the ability to degrade osmolytes, aromatics, and chlorophyll in marine bacterial genomes, and chemotactic and low-oxygen adaptive abilities in terrestrially sourced bacterial genomes. This evolutionary strategy suggests that organisms from these proteobacterial lineages can flexibly modify their gene repertoire in response to substrate and energy conditions. The novel Proteobacteria community discovered in the hydrothermal ecosystem are not made up of highly-adapted microbial lineages that are only limited in this environmental setting, but rather of universally distributed lineages that have adopted strategies to live in these environments. Our findings call for quantifying the wide ecological impacts of these proteobacterial lineages. Overall, our approach will facilitate further investigations on links between genome diversification patterns and functional ecology in other microbial groups and environments.

## Supplementary information

Supplementary Figure S1

Supplementary Figure S2

Supplementary Figure S3

Supplementary Figure S4

Supplementary Figure S5

Supplementary Table S1

Supplementary Table S2

Supplementary Table S3

Supplementary Table S4

Supplementary Table S5

Supplementary Table S6

Supplementary Table S7

Supplementary Table S8

Supplementary Table S9

Supplementary Table S10

Supplementary Table S11

## Data Availability

Raw metagenome and metatranscriptome sequence reads are deposited in NCBI BioProject database with the accession numbers of PRJNA314399, PRJNA283159, PRJNA234377, PRJNA72707, and PRJNA283173; for detailed accession numbers refer to Supplementary Table S1. NCBI Genbank accession numbers for individual genomes could be found under the BioProject ID PRJNA522654 and in Supplementary Table S2. Additional detailed annotation results for individual genomes are available from the corresponding author on request.
