# Supplementary material for: Genome diversification in globally distributed novel marine Proteobacteria is linked to environmental adaptation
Source: ISME J. 2020 May 11;14(8):2060–77. doi: 10.1038/s41396-020-0669-4 (PMC7367891; doi:10.1038/s41396-020-0669-4)
Supplement: Supplementary file 5 — Supplementary Figure S5 [file 41396_2020_669_MOESM5_ESM.pdf]

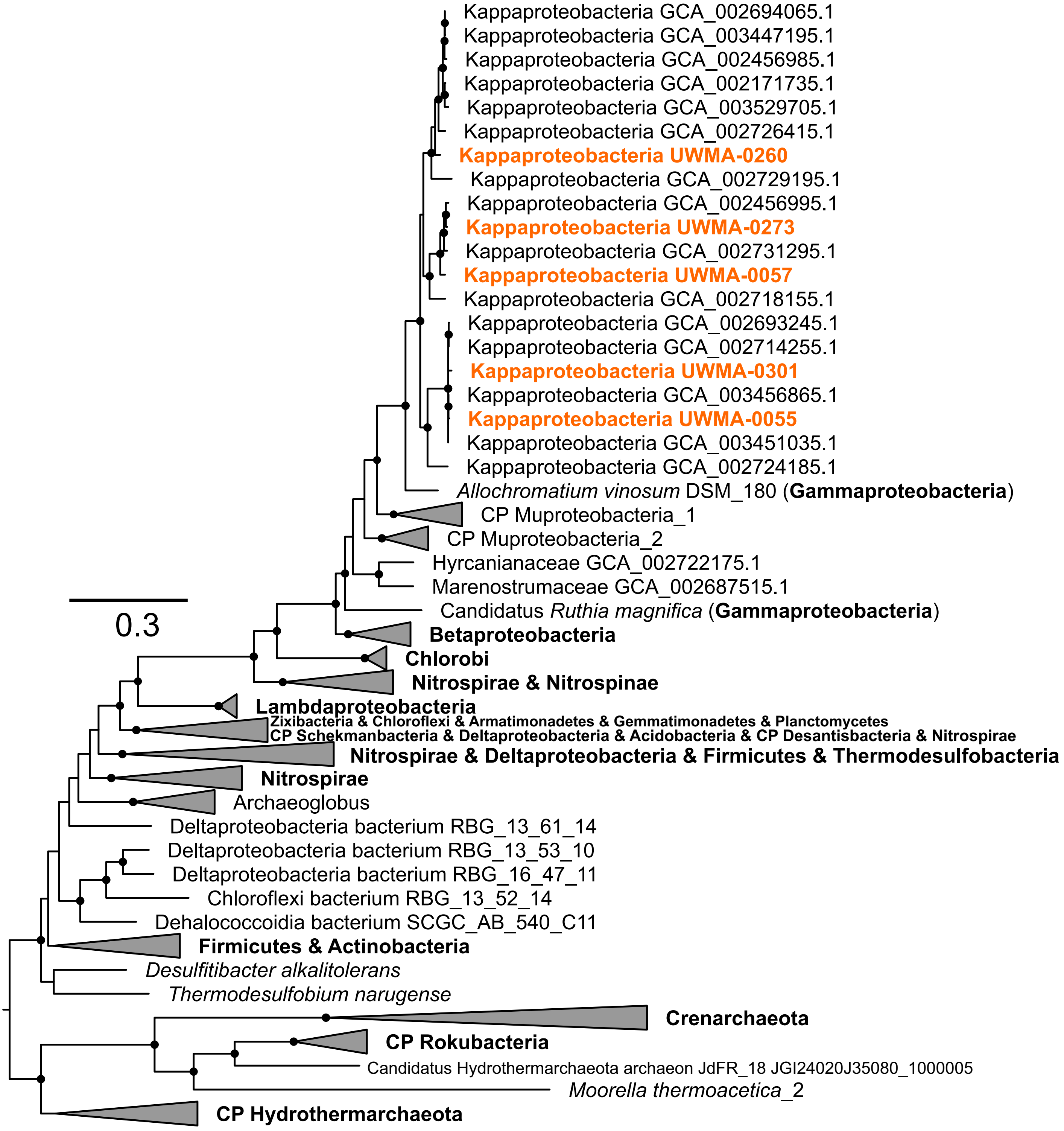

Figure S5. Concatenated phylogenetic tree of *dsrAB* proteins. DsrA and DsrB proteins were aligned with reference sequences independently and concatenated. The concatenated protein alignment was trimmed with gapthreshold of 25% using trimAl v1.2. The phylogenetic tree was reconstructed by IQ-TREE v1.6.9 with settings as described in the methods. Branches with over 90% UFBoot bootstrap values were labeled with closed circles. Genomes from this study were highlighted in bold.
